# FDA-approved PDE4 inhibitors alleviate the dominant toxicity of ALS-FTD-associated CHCHD10^S59L^ in *Drosophila* and human cells

**DOI:** 10.1016/j.isci.2026.114879

**Published:** 2026-02-02

**Authors:** Swati Maitra, Do-Won Ham, Minwoo Baek, Yun-Jeong Choe, Nam Chul Kim

**Affiliations:** 1 Department of Pharmacy Practice and Pharmaceutical Sciences, College of Pharmacy, University of Minnesota, Duluth, MN 55812, USA

**Keywords:** Pharmacology, Entomology, Molecular biology, Neuroscience

## Abstract

Mutations in CHCHD10 are a genetic cause of ALS-FTD. In our previous studies using *Drosophila* expressing C2C10H^S81L^ and human cells expressing CHCHD10^S59L^, we found that the aberrant activation of the PINK1/Parkin pathway drives cellular toxicity, and pseudo-substrate inhibitors of PINK1 or mitofusin-2 agonists can mitigate these effects. Evidence from *in vitro*, *in vivo*, and chemical approaches supports PINK1 inhibition as a promising strategy for CHCHD10^S59L^-associated disease. Here, we show that FDA-approved PDE4 inhibitors significantly reduce CHCHD10^S59L^-induced mitochondrial morphological and functional defects in both human cells and *Drosophila*. These protective effects occur through a cAMP/PKA-dependent mechanism, indicating that elevated cAMP signaling attenuates aberrant PINK1/Parkin activation. Moreover, forskolin combined with PDE4 inhibitors synergistically decreases mitochondrial toxicity at lower concentrations. Together, our findings suggest that clinically available PDE4 inhibitors could be repurposed for CHCHD10^S59L^-linked ALS-FTD, while emphasizing the need to carefully consider the effects of the PINK1/Parkin pathway, as it is generally recognized as a protective pathway.

## Highlights

• PDE4 inhibitors reduce CHCHD10^S59L^ mitochondrial defects
• Protective effects are cAMP/PKA dependent and act via PINK-Parkin pathway
• Low-doses of forskolin and PDE4 inhibitor provide synergistic boost
• PDE4 inhibitors can be repurposed for ALS-FTD via PINK1/Parkin pathway

## Graphical abstract

**Figure:**
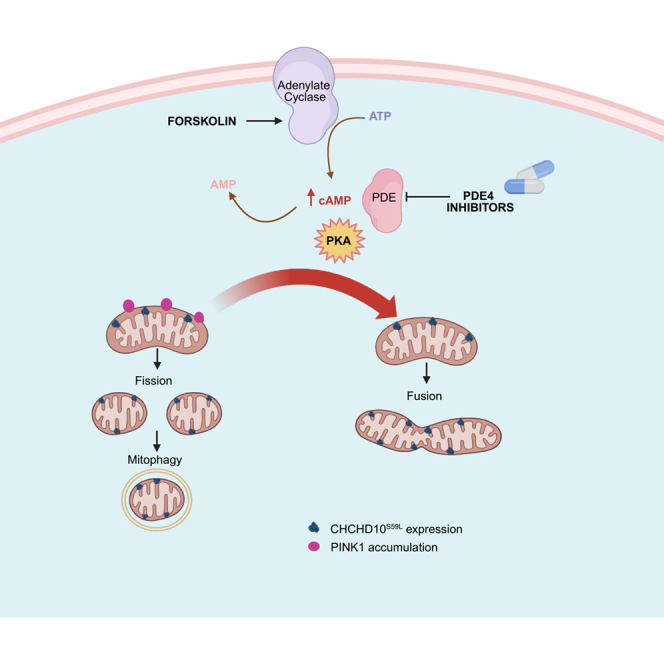

## Introduction

Coiled-coil-helix-coiled-coil-helix domain containing 10 (*CHCHD10*) encodes a mitochondrial protein that causes multiple different degenerative diseases, including amyotrophic lateral sclerosis and/or frontotemporal dementia (ALS-FTD), when it is mutated.^1^ Growing evidence has linked the maintenance of balanced mitochondrial dynamics to the preservation of neuronal health and the support of the complex functional demands of highly adaptable neuronal networks.^2^ In a previous study from our laboratory, we demonstrated that the overexpression of a mutant CHCHD10 harboring the S59L substitution in human cell line models of ALS-FTD disrupted mitochondrial dynamics, resulting in pronounced mitochondrial fragmentation and functional impairment. Consistently, expressing C2C10H^S81L^ (*Drosophila* homolog of human CHCHD2 and CHCHD10 bearing a mutation in the conserved position with human S59L) with the tissue-specific GAL4/UAS expression system, induced rough eye phenotypes, neuromuscular junction defects, and muscle degeneration with fragmented mitochondria.^3^ Through a series of genetic interaction studies, we identified that PTEN-induced kinase 1 (PINK1) and Parkin, two key regulators of mitochondrial quality control and mitophagy,^4^ are strong modifiers for the dominant toxicity of C2C10H^S81L^ in *Drosophila*. RNAi-mediated knockdown of PINK1 and Parkin strongly suppressed the rough eye phenotypes, muscle degeneration, and mitochondrial fragmentation of C2C10H^S81L^. Additionally, ATP production was recovered in the adult indirect flight muscles. In contrast, overexpression of PINK1 and Parkin significantly enhanced the rough eye phenotypes.^3^

In human cell models such as HeLa and SH-SY5Y cells, the expression of the CHCHD10^S59L^ led to the accumulation of PINK1 and Parkin. This was followed by the increased activation of LC3 (microtubule-associated protein 1A/1B-light chain 3), a key marker of autophagy and mitophagy.^3^ Consistent with the results from the *Drosophila* system, the reduction of PINK1 and Parkin expression through RNAi (siRNA) substantially reduced mitochondrial fragmentation induced by CHCHD10^S59L^ and also improved functional mitochondrial respiration. Furthermore, knocking down the mitophagy adaptors for the PINK1/Parkin pathway showed recovery of functional mitochondrial respiration in both the cell lines. Significantly, siRNA-mediated knockdown of PINK1 reversed the mitochondrial network fragmentation in two patient-derived fibroblasts.^3^ Therefore, these results suggest that CHCHD10^S59L^ induces excessive or dysregulated mitophagy, and the PINK1/Parkin pathway plays a pivotal role in the pathophysiology of CHCHD10^S59L^-induced diseases, and that the modulation of this pathway could present a therapeutic avenue for mitigating CHCHD10^S59L^-induced diseases. In fact, we identified two pseudo-substrate inhibitors for PINK1 and demonstrated that two peptide inhibitors reduced its ubiquitin kinase activity and attenuated CHCHD10^S59L^-induced mitochondria fragmentation.^3^ One of the major hurdles in targeting the PINK1/Parkin pathway therapeutically is the lack of small-molecule inhibitors for PINK1, making it difficult to assess the efficacy of PINK1 inhibition *in vivo*. In search of potential therapeutic strategies, we turned to a pharmacological approach where we sought small molecules that may directly or indirectly reduce PINK1 expression levels or enzymatic activity.

In 2011, Merrill et al.^4^ demonstrated that a mitochondria-localized A kinase anchoring protein 1 (AKAP1) associates with cAMP-dependent protein kinase A (PKA) to the outer mitochondrial membrane (OMM) and phospho-inhibits the fission protein dynamin related protein 1 (Drp1). The forskolin treatment (adenylate cyclase activator) elevated cAMP and favored mitochondrial fusion over fission, thereby contributing to mitochondrial integrity and offering neuroprotection. The expression of OMM targeted form of PKA reformed the mitochondrial reticulation, and the OMM targeted form of PKA inhibitor promoted mitochondrial fragmentation. They reported the principal mechanism underlying the OMM anchored PKA-AKAP1 complex, which promotes mitochondrial elongation, was via site-specific phosphorylation (Ser637) of Drp1, stabilizing it as an incompatible oligomeric complex with the fission process. These findings highlighted the potential of targeting kinase signaling at mitochondria to remodel mitochondrial dynamics for therapeutic benefit in neurodegenerative conditions.^4^

Interestingly, another study by Akabane et al. in 2016,^5^ demonstrated that PKA activation by elevated cAMP levels with forskolin treatment regulates the stability of PINK1 and impairs Parkin recruitment to damaged (depolarized) mitochondria. This process occurred through phosphorylating mitofilin (MIC60) by PKA, one of the core components of the mitochondrial contact site and cristae organizing system (MICOS) complex. Based on these reports, we hypothesized that augmenting cAMP levels might serve as a promising strategy to reduce the toxicity of CHCHD10^S59L^ by modulating the PINK1-Parkin pathway indirectly.

The intracellular concentration of cAMP is maintained by complex homeostatic mechanisms that encompass adenylyl cyclases (which catalyze the conversion of AMP into 3′–5′ cAMP) and phosphodiesterases (PDE; which catalyze the conversion of cyclicnucleotides to AMP). PDE inhibitors have shown to increase cAMP levels, thus indicating a promising avenue for drug development due to their ability to modulate a broad spectrum of biological processes, including memory, metabolism, and cell survival, and so forth.^6^^,^^7^ Therefore, we assessed the efficacies of PDE4 inhibitors along with forskolin to observe any meaningful biological effects on the key pathway involved in the CHCHD10^S59L^-mediated mitochondrial pathology. Our findings indicate that FDA-approved PDE4 inhibitors mitigate CHCHD10^S59L^(C2C10H^S81L^)-induced toxicity in *Drosophila* and human cells by reducing the PINK1/Parkin pathway in a cAMP/PKA-dependent manner.

## Results

### Forskolin blocks PTEN-induced kinase 1 and Parkin accumulation

**Figure 1:**
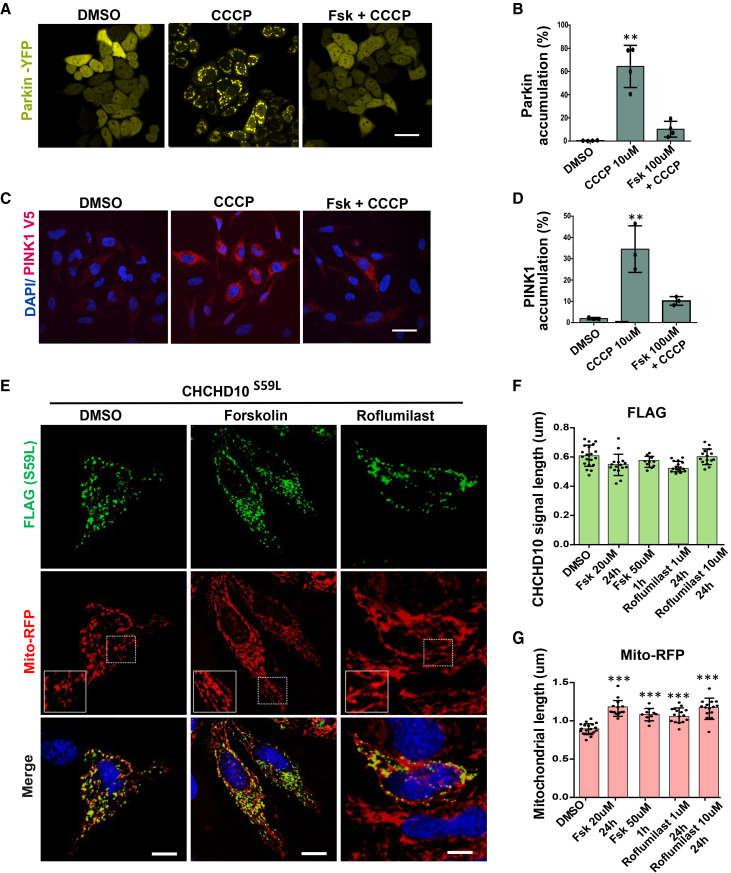
Forskolin blocks PINK1-Parkin accumulation and mitigates CHCHD10^S59L^-induced cellular toxicity HeLa ^YFP−Parkin^ cells and HeLa ^PINK1-V5−His^ cells were pre-treated with forskolin (100 μM-2h) followed by CCCP (10 μM-1 h). (A) Immunohistochemical images for the visualization of mitochondrial Parkin-YFP accumulation in DMSO, CCCP, and Fsk+CCCP groups, respectively. Scale bars, 50uM. (B) Graphical representation indicates the percentage of mitochondrial Parkin accumulation in cells. (C) Immunohistochemical images for the visualization of PINK1 accumulation in cells, immune-stained with anti-Pink1 antibody (red). Scale bars, 50um. (D) Graphical representation indicates the percentage of mitochondrial PINK1 accumulation in cells. Each independent experiment includes>200 cells for each group. Data are shown as mean ± SD (one-way ANOVA with *post hoc* Dunnett’s Test). (E) HeLa cells were transfected with FLAG-tagged CHCHD10^S59L^ in the DMSO, forskolin, and roflumilast-containing medium. Cells were infected with Mito-RFP (Red, mitochondria) for overnight. Representative images of cells immunostained with anti-FLAG antibody (green, CHCHD10^S59L^), Mito-RFP, and DAPI. Scale bars, 20 μM. (F and G) Quantification of CHCHD10-FLAG (green) and Mito-RFP (red) signal length. Data are shown as mean ± SD (one-way ANOVA and *post hoc* Dunnett’s test, two-sided comparison with DMSO, ∗∗∗*p* < 0.001, ∗∗*p* < 0.01, and ∗*p* < 0.05).

In order to validate the findings of Akabane et al., we tested whether forskolin, an adenylate cyclase activator, reduces PINK1 and Parkin accumulation induced by CCCP. CCCP, a mitochondrial uncoupler, has been shown to induce PINK1 stabilization and subsequent Parkin accumulation on mitochondria.^8^ As demonstrated,^5^ acute forskolin (100 μM) treatment almost completely blocked CCCP-induced Parkin accumulation in HeLa cells stably expressing Parkin-YFP (Figures 1A and 1B). Concurrently, we demonstrated that forskolin treatment also blocked PINK1 accumulation in HeLa cells stably expressing PINK1 tagged with V5 (Figures 1C and 1D). These observations guided us to further investigate the effects of forskolin on CHCHD10^S59L^-induced mitochondrial toxicity.

### Forskolin and roflumilast mitigate the toxicity of coiled-coil-helix-coiled-coil-helix domain containing 10^S59L^ in cell models

To determine whether the modulation of the cAMP pathway can alleviate mitochondrial toxicity induced by CHCHD10^S59L^, when we treated forskolin to HeLa cells transiently transfected with CHCHD10^S59L^, mitochondrial branch length increased compared to that of DMSO-treated cells (Figure 1E). Given forskolin’s established mechanism of increasing cyclic AMP levels through the activation of adenylate cyclase, we investigated the potential of PDE4 inhibitor to elicit a similar effect by preventing cAMP degradation. First, we chose roflumilast, a selective PDE4 inhibitor that was approved and marketed for the treatment of chronic obstructive pulmonary disease (COPD).^9^^,^^10^ When we treated CHCHD10^S59L^-expressing HeLa cells with roflumilast (1 μM or 10 μM for 24 h), mitochondrial fragmentation caused by CHCHD10^S59L^ was reduced while the aggregation of CHCHD10^S59L^ protein was not affected (Figures 1E–1G).

### Cyclic adenosine monophosphate-protein kinase A signaling mediates the mitochondrial protective effects of forskolin and roflumilast

**Figure 2:**
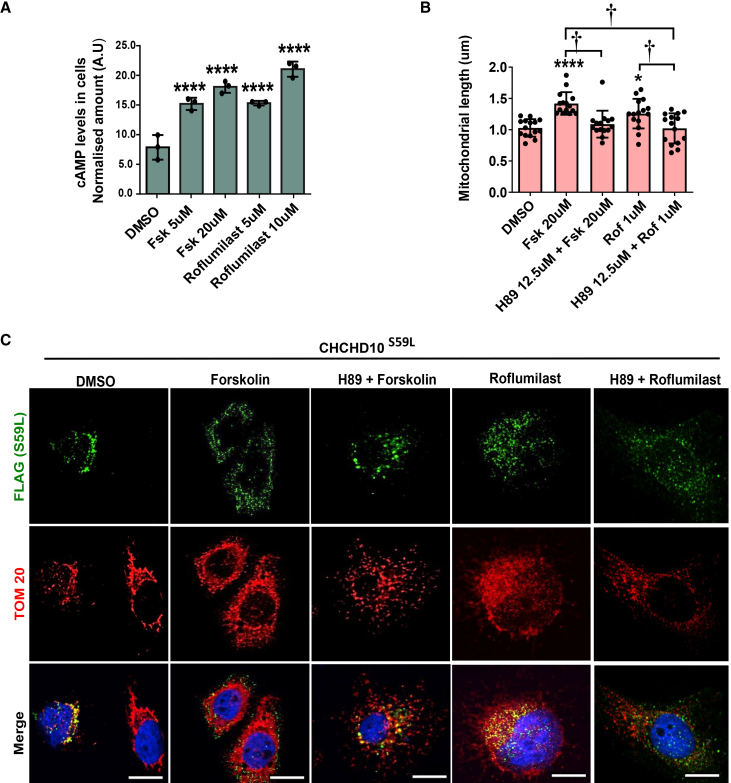
cAMP-PKA signaling mediates the mitochondrial protective effects of Forskolin and Roflumilast HeLa cells were transfected with CHCHD10^S59L^ and treated with drugs, respectively. (A) Graphical representation of cAMP levels in HeLa cells expressing the CHCHD10^S59L^ mutant, along with drug treatment. Data are shown as mean ± SD (one-way ANOVA and *post hoc* Dunnett's test, two-sided comparison with DMSO, ∗∗∗*p* < 0.001, ∗∗*p* < 0.01, and ∗*p* < 0.05). (B) Quantification of mitochondrial length in CHCHD10^S59L^-transfected HeLa cells treated with Forskolin or Roflumilast, with or without the PKA inhibitor H89. H89 pre-treatment (12.5 μM-4 h) abolished the protective effects of Forskolin (20 μM-24 h) and Roflumilast (1 μM-24 h) (∗*p* < 0.05, ∗∗∗∗*p* < 0.0001 vs. DMSO; †*p* < 0.05 between indicated groups; one-way ANOVA with *post-hoc* Tukey’s test). (C) Representative confocal images of cells stained with anti-FLAG (green, CHCHD10^S59L^), anti-TOM20 (red, mitochondria), and DAPI (blue, nuclei). Scale bars, 20 μm.

To explore whether forskolin and roflumilast act through the canonical cAMP-PKA signaling axis, we measured cAMP levels and used a PKA inhibitor, H89. All drug-treated groups exhibited a significant elevation in intracellular cAMP levels compared to the control group. (Figure 2A). Pharmacological inhibition of PKA using H89 (12.5 μM) (Figures 2B, 2C, S1A, and S1B) indicates that the activation of the cAMP-PKA pathway is necessary for the morphological rescue conferred by forskolin and roflumilast.

### Forskolin and roflumilast reduce PTEN-induced kinase 1 accumulation caused by coiled-coil-helix-coiled-coil-helix domain containing 10^S59L^ in HeLa^PINK1-V5^ cells

**Figure 3:**
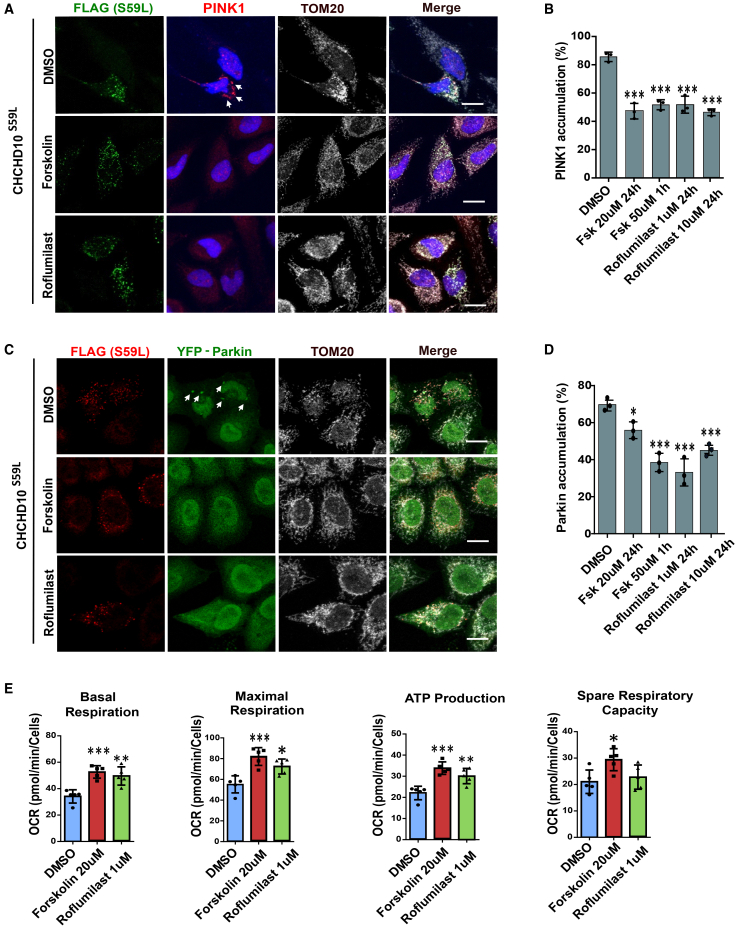
Forskolin and Roflumilast reduce PINK1-Parkin accumulation induced by CHCHD10^S59L^ expression in HeLa cells HeLa^PINK1-V5^ cells and HeLa^YFP−Parkin^ cells were transfected with FLAG-tagged CHCHD10^S59L^ in the DMSO, Forskolin, and Roflumilast-containing medium. (A) Representative images of CHCHD10^S59L^ expressing mitochondria cultured in DMSO, Forskolin, and Roflumilast-containing media. Cells were immunostained with anti-FLAG (green, CHCHD10^S59L^) and anti-PINK1 (red, PINK1) and anti-TOM20 (white, mitochondria) antibodies. Scale bars, 20 μM. (B) Bar graphs indicate the percentage of HeLa^PINK1-V5^ showing PINK1 accumulation in CHCHD10^S59L^ expressing mitochondria. Data are shown as mean ± SD (one-way ANOVA and *post hoc* Dunnett’s test, two-sided, comparison with DMSO). (C) Representative images from CHCHD10^S59L^ express mitochondria cultured in DMSO, Forskolin (20 μM), and Roflumilast (1 μM) containing media. Cells were immunostained against FLAG (green, CHCHD10^S59L^) and TOM20 (white, mitochondria) antibodies. Arrows indicate accumulated parkin (green, YFP-parkin) in the mitochondria. Scale bars, 20 μM. (D) Bar graphs indicate the percentage of HeLa^YFP−Parkin^ showing Parkin accumulation in CHCHD10^S59L^ expressing mitochondria. Data are shown as mean ± SD (one-way ANOVA and *post hoc* Dunnett's test, two-sided, comparison with DMSO. (E) Mitochondrial respiration was measured by Seahorse XF Cell Mito Stress tests 24 hrs after CHCHD10^S59L^ transfection in DMSO, Forskolin, and Roflumilast-treated HeLa cells. Data are shown as mean ± SD (one-way ANOVA and *post hoc* Dunnett’s test, two-sided, comparison with DMSO, ∗∗∗*p* < 0.001, ∗∗*p* < 0.01, and ∗*p* < 0.05).

As we previously reported, CHCHD10^S59L^ expression induced PINK1 accumulation on mitochondria in an uncontrolled manner. A reduction of PINK1 accumulation or activity has been shown to be morphologically and functionally beneficial to mitochondria.^3^ Consequently, we investigated whether Forskolin and Roflumilast would reduce CHCHD10^S59L^-induced PINK1 accumulation in HeLa cells. For this purpose, we used HeLa cells stably expressing V5-tagged PINK1 (HeLa^PINK1-V5^ cells).^11^ Following the transfection and expression of CHCHD10^S59L^, more than 80% of the cells exhibited PINK1 accumulation on mitochondria, and the PINK1 aggregation-positive cells were reduced by forskolin and roflumilast treatment (Figures 3A and 3B). Acute treatment of 50 μM forskolin and 24-hour-treatment of 20 μM forskolin exhibited similar effects (Figure 3B). However, we did not observe dose-dependent effects of roflumilast when it was treated with 1 μM or 10 μM concentrations in this experiment (Figure 3B).

### Forskolin and roflumilast reduce Parkin accumulation caused by coiled-coil-helix-coiled-coil-helix domain containing 10^S59L^ in HeLa^Parkin−YFP^ cells

Mitochondria-localized PINK1 phosphorylates ubiquitins and the ubiquitin-like domain of Parkin, resulting in Parkin accumulation on mitochondria.^12^ Therefore, we investigated whether forskolin and roflumilast reduce Parkin accumulation in HeLa cells via reducing CHCHD10^S59L^-induced PINK1 accumulation. For this purpose, we used HeLa cells stably expressing yellow fluorescent protein (YFP)-tagged Parkin. When CHCHD10^S59L^ was transfected and expressed, approximately 70% of the cells had Parkin-YFP puncta that colocalized with mitochondria (Figures 3C and S3A). Although acute treatment with 50 μM forskolin reduced Parkin-YFP accumulation, treatment with 20 μM forskolin for 24 h treatment showed a more significant decrease in Parkin accumulation on mitochondria (Figure 3D). Interestingly, a relatively low dose of roflumilast (1 μM) reduced Parkin-YFP accumulation more than 10 μM Roflumilast (Figure 3D). This may be due to the dynamic regulation of cytosolic cAMP levels by cAMP-activated PKA and PKA-activated phosphodiesterase^13^ or a near-maximal inhibition and cAMP elevation at low concentrations.^13^ To determine whether these morphological improvements were accompanied by functional recovery, we next assessed mitochondrial bioenergetics through Seahorse assay. Consistently, the analysis revealed that forskolin and/or roflumilast treatment increased basal mitochondrial respiration and ATP production with increased maximal respiration (Figure 3E). There was also a minimal increase in the spare capacity of samples treated with roflumilast (Figure 3E). Furthermore, in SH-SY5Y neuroblastoma cells transiently co-transfected with CHCHD10^S59L^ and mCherry-Parkin, treatment with forskolin or roflumilast similarly reduced Parkin accumulation on mitochondria, consistent with observations in HeLa cells. The activation of the cAMP-PKA pathway thus appears to suppress aberrant Parkin recruitment in both neuronal and non-neuronal contexts. Seahorse analysis in SH-SY5Y cells revealed that forskolin and roflumilast treatment enhanced mitochondrial respiration in CHCHD10^S59L^-expressing SH-SY5Y cells, as indicated by increased maximal and spare respiratory oxygen consumption rates (Figures S2B and S2C).

### Forskolin and roflumilast reduced LC3 accumulation on mitochondria

**Figure 4:**
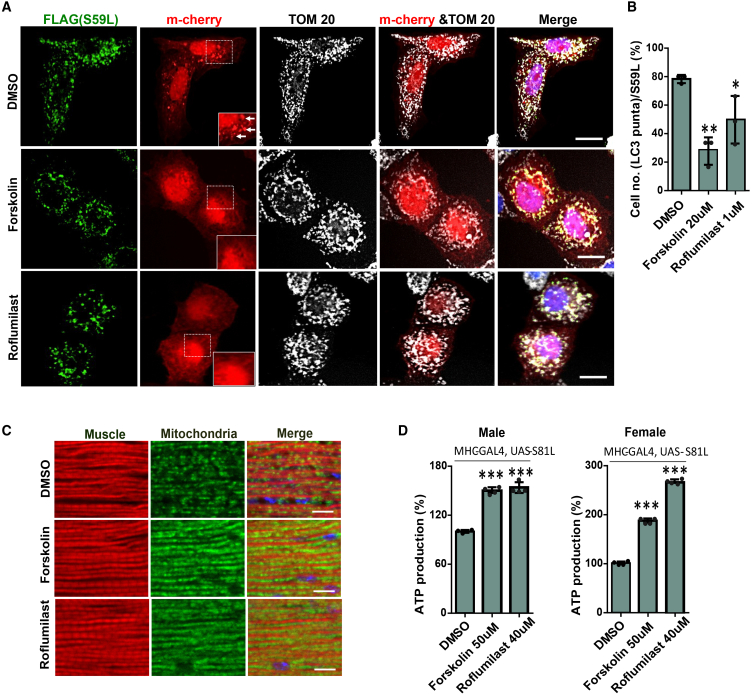
Forskolin and Roflumilast alleviate mitophagy and muscle damage in *Drosophila* HeLa^YFP−Parkin^ cells were co-transfected with FLAG-tagged CHCHD10^S59L^ and mCherry-LC3 in the DMSO, Forskolin, and Roflumilast-containing medium. (A) Representative images of CHCHD10^S59L^ expressing mitochondria cultured in DMSO, Forskolin (20 μM), and Roflumilast (1 μM) containing media. Cells were immunostained with anti-FLAG (green, CHCHD10^S59L^) and anti-TOM20 (white, mitochondria) antibodies. Arrows indicate LC3 puncta in the CHCHD10^S59L^ expressing mitochondria. Scale bars, 20uM. (B) Graphical quantification of the percentage of HeLa^YFP−parkin^ shows LC3-mcherry puncta forming in the CHCHD10^S59L^ expressing mitochondria. Data are shown as mean ± SD (one-way ANOVA and *post hoc* Dunnett’s test, two-sided, comparison with DMSO). (C) Representative micrographs of indirect flight muscles from 10-day-old adult flies (MHC-Gal4, UAS-S81L) fed with Forskolin (50 μM) and Roflumilast (40 μM), immunostained with streptavidin-Alexa Fluor 488 and phalloidin-Alexa Fluor 594. DMSO was used as a vehicle. Scale bars, 10uM. (D) Graphical representation of ATP levels in thoraxes from 10-day-old flies using four independent replicates for males and females, respectively. Data are shown as mean ± SD (one-way ANOVA and *post hoc* Dunnett’s test, two-sided, ∗∗∗*p* < 0.001, compared to DMSO).

The PINK1/Parkin signaling pathway has been demonstrated to regulate mitophagy.^12^ We have shown previously that CHCHD10^S59L^ expression resulted in increased LC3 accumulation and subsequent mitophagy.^3^ Thus, we investigated whether forskolin and roflumilast could mitigate CHCHD10^S59L^-induced LC3 accumulation. Using HeLa cells stably expressing YFP-Parkin and transient co-transfection with CHCHD10^S59L^ and LC3, we observed that nearly 80% of the CHCHD10^S59L^-transfected HeLa YFP-Parkin cells showed LC3 accumulation, and forskolin and roflumilast treatment reduced LC3 accumulation (Figures 4A and 4B).

### Forskolin and roflumilast ameliorated mitochondrial defects induced by C2C10H^S81L^ in *Drosophila*

To validate our findings at the systems level, we investigated the effect of forskolin and roflumilast treatment on mitochondrial defects in *Drosophila* expressing C2C10H^S81L^. For this, we expressed C2C10H^S81L^ in muscle tissues with the MHC-GAL4 driver. The C2C10H^S81L^ flies were reared in fly food containing forskolin (50 μM), roflumilast (40 μM), and DMSO. Indirect flight muscle sections of C2C10H^S81L^ flies stained with a fluorophore-conjugated streptavidin (green) and phalloidin (red) revealed fragmented mitochondria along with muscular degeneration (Figure 4C). This condition was ameliorated in forskolin and roflumilast-treated flies (Figures 4C, S3B, and S3C). ATP levels were measured as an indicator of mitochondrial dysfunction in the fly thoraxes, which primarily consist of muscle tissues. We observed improved ATP levels in the muscle tissues of flies expressing C2C10H^S81L^ treated with forskolin and roflumilast, as compared to DMSO-fed C2C10H^S81L^ flies (Figure 4D). Therefore, forskolin and roflumilast imparted a rescue effect on the mitochondrial defects induced by toxic C2C10H^S81L^.

### Other phosphodiesterase 4 inhibitors, apremilast and rolipram, are also effective

**Figure 5:**
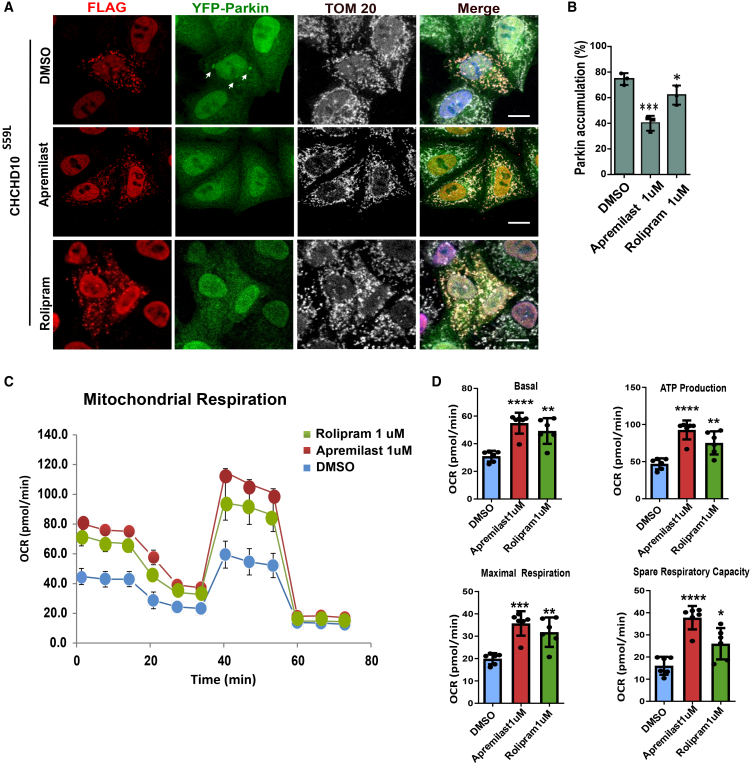
Effects of other PDE4 inhibitors on abnormal mitochondrial phenotypes (A) Representative images from CHCHD10^S59L^ expressing mitochondria cultured in DMSO, Apremilast (1 μM), and Rolipram (1 μM) containing media in HeLa cells. Cells were immunostained against anti- FLAG (red, CHCHD10^S59L^) and anti-TOM20 (white, mitochondria) antibodies. Arrows indicate accumulated parkin in the mitochondria. Scale bars, 20uM. (B) Graphical representation indicates the percentage of HeLa showing Parkin-YFP accumulation in CHCHD10^S59L^ expressing mitochondria. Data are shown as mean ± SD (one-way ANOVA and *post hoc* Dunnett’s test, two-sided, comparison with DMSO, ∗∗∗*p* < 0.001, ∗∗∗*p* < 0.01, and ∗*p* < 0.05). (C and D) Mitochondrial Respiration by Seahorse XF Cell Mito Stress tests 24 hrs after CHCHD10^S59L^ transfection in DMSO, Apremilast, and Rolipram treated HeLa cells. Data are shown as mean ± SD (one-way ANOVA and *post hoc* Dunnett’s test, two-sided, comparison with DMSO, ∗∗∗*p* < 0.001, ∗∗*p* < 0.01, and ∗*p* < 0.05).

To further validate the potential of PDE4 inhibitors against CHCHD10^S59L^-mediated toxicity, we also tested a first-generation PDE4 inhibitor, rolipram,^14^^,^^15^ and another approved drug, apremilast, which is prescribed for plaque psoriasis or psoriatic arthritis.^16^^,^^17^ Both apremilast and rolipram effectively reduced Parkin-YFP accumulation in HeLa^YFP−Parkin^ cells transiently transfected with CHCHD10^S59L^ (Figure 5A). However, rolipram showed only marginal efficacy, though the amelioration was reproducible in several independently repeated experiments (Figure 5B). The seahorse analysis further verified that both apremilast and rolipram reduced the toxicity of CHCHD10^S59L^, while apremilast was more effective than rolipram in rescuing defective mitochondrial function caused by CHCHD10^S59L^ (Figures 5C and 5D). A similar trend in mitochondrial respiration was also reflected in the seahorse assay using SH-SY5Y neuroblastoma cells (Figures S2D and S2E). To obtain an overall understanding, Seahorse assays in HeLa cells expressing CHCHD10^S59L^ showed that treatment with Forskolin (20 μM) and the PDE4 inhibitors, Roflumilast, Apremilast, and Rolipram (each at 1 μM), significantly enhanced mitochondrial ATP production compared to the DMSO control, demonstrating their capacity to improve mitochondrial function (Figures S4A–S4C).

### Evaluation of mitophagy using the mito-QC reporter system

**Figure 6:**
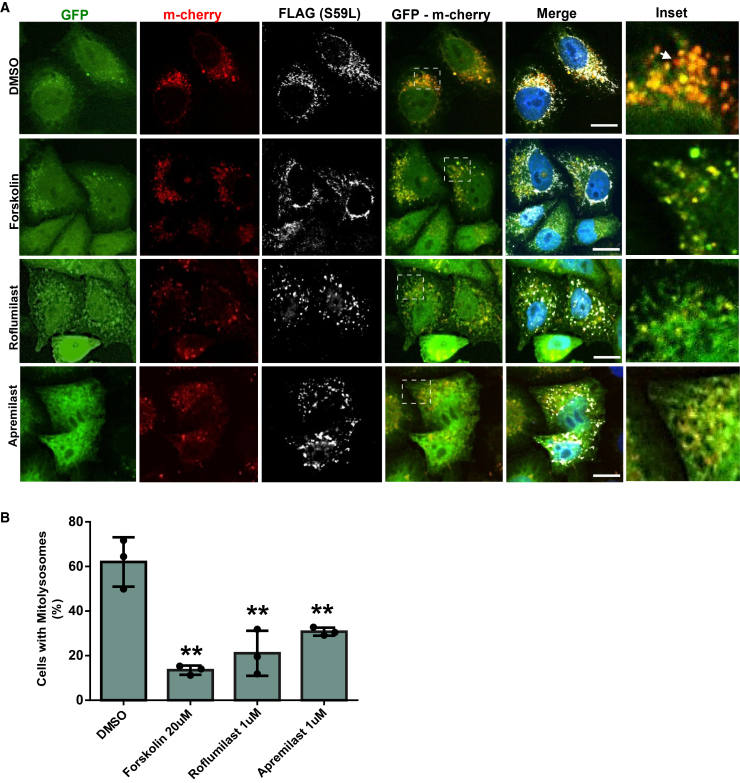
Evaluation of mitophagy using the mito-QC reporter in CHCHD10^S59L^-expressing cells Hela^Parkin−YFP^ cells were co-transfected with CHCHD10, the mito-QC reporter system, and treated with the respective drug or DMSO. (A) Representative confocal images of CHCHD10^S59L^-expressing HeLa cells transfected with the mito-QC reporter plasmid. Cells were treated with vehicle (DMSO), Forskolin (20 μM), Roflumilast (1 μM), or Apremilast (1 μM) for 24 h. Mitophagy events are visualized by mitolysosomes (mCherry^+^/GFP^−^ puncta-arrows in inset). (B) Graphical quantification of cells with mitolysosomes (%). PDE4 inhibitor treatment significantly decreased mitophagy compared with DMSO controls (*p* < 0.05, one-way ANOVA followed by *post hoc* Dunnett’s multiple comparison test; ∗*p* < 0.05, ∗∗*p* < 0.01 vs. DMSO). Data represented here mean ± SD from three independent experiments. Scale bars, 20 μm.

Since CHCHD10^S59L^ expression leads to activated PINK1/Parkin pathway and mitophagy, we next examined whether PDE4 inhibition could attenuate this process using the mito-QC reporter system. Treatment with PDE4 inhibitors significantly reduced mitophagy flux compared to vehicle-treated controls (Figures 6A and 6B) in CHCHD10^S59L^ expressing HeLa^Parkin−YFP^ cells, consistent with LC3 accumulation (Figures 4A and 4B). Interestingly, among the compounds tested, Apremilast did not produce the strongest reduction in mitophagy (no statistically significant difference between these groups), despite being the most effective in enhancing mitochondrial respiration as assessed by Seahorse analysis (Figures S4A–S4C). These results indicate that the suppression of mitophagy alone is insufficient to restore cellular energy metabolism. Instead, additional PINK1-dependent but Parkin-independent mechanisms likely contribute to the full recovery, consistent with our previous findings.^3^

### Enhanced mitochondrial recovery through the synergistic action of forskolin and phosphodiesterase 4 inhibitors

**Figure 7:**
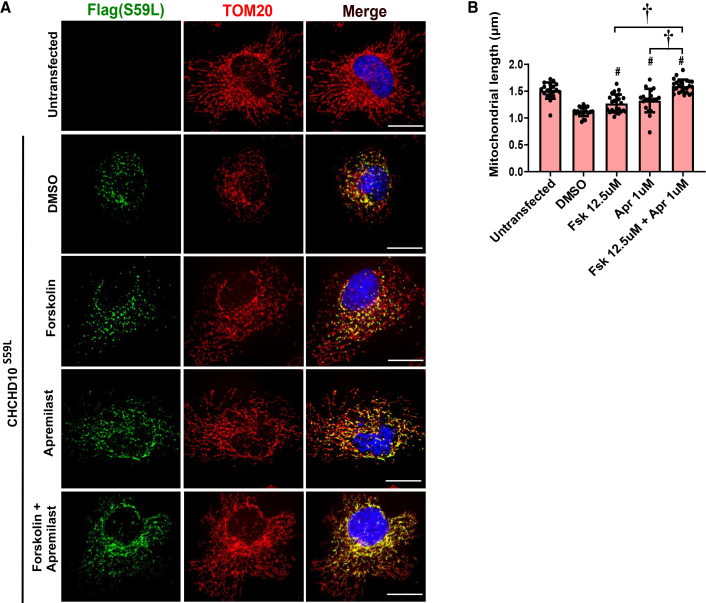
Additive effects of Forskolin and PDE4 inhibitors on mitochondrial network integrity in CHCHD10^S59L^-transfected HeLa cells HeLa cells were transfected with FLAG-tagged CHCHD10^S59L^ and treated with Forskolin (12.5 μM) and Apremilast (1 μM) or both for 24 h. (A) Representative images of cells immunostained with anti-FLAG (green, CHCHD10^S59L^), anti-TOM20 (Red, mitochondria), and DAPI (blue, nuclei). Scale bars, 20 μm. (B) Quantification of mitochondrial length. Combined treatment with Forskolin and Apremilast significantly restored mitochondrial network integrity compared with single-drug treatments (*p* < 0.05, one-way ANOVA followed by *post hoc* Tukey’s test). Data are presented as mean ± SD (#*p* < 0.05 vs. DMSO; †*p* < 0.05 between indicated groups.).

Given the individual efficacy of forskolin and various PDE4 inhibitors in mitigating CHCHD10^S59L^-induced mitochondrial toxicity, we finally examined whether combined cAMP modulation could produce additive or synergistic protective effects. To address this, we examined two complementary models of mitochondrial damage: a CCCP-induced Parkin accumulation model and a CHCHD10^S59L^-expressing cell model. When HeLa^Parkin–YFP^ cells were pre-treated with Forskolin (50 μM) together with either Roflumilast (1 μM) or Apremilast (1 μM) to elevate intracellular cAMP levels, followed by CCCP treatment, a significant reduction in Parkin accumulation was observed compared to each single-drug treatment condition (Figures S5A–S5D). This additive or potentially synergistic effect appeared to be more prominent with the Forskolin and Apremilast combination (Figures S5C and S5D). Furthermore, we confirmed that this additive or synergistic effect also produced a significant protective effect against mitochondrial fragmentation caused by CHCHD10^S59L^, in which the disrupted mitochondrial network structure was substantially restored by the combined treatment of Forskolin and Apremilast compared to each single-drug treatment (Figures 7A and 7B). To further examine the robustness of this combinatorial effect, we tested whether the protective effect could still be observed under reduced drug concentrations. When Forskolin and Apremilast were applied at lower doses (5 μM and 0.5 μM, respectively), every single treatment alone failed to rescue the CHCHD10^S59L^-induced mitochondrial defects. However, their combined treatment markedly restored the mitochondrial network structure, indicating that the synergistic interaction between Forskolin and Apremilast remains effective even at submaximal concentrations (Figures S5E and S5F). These results imply that targeting cAMP homeostasis through dual mechanisms via stimulating its production and blocking its breakdown provided a more effective therapeutic strategy against CHCHD10^S59L^-associated mitochondrial toxicity than either approach alone.

## Discussion

Mitochondrial dysfunction remains one of the prominent pathological features of neurodegenerative disorders, including ALS-FTD.^18^ Emerging reports suggest that cAMP/PKA signaling plays a key role in mitochondrial integrity and homeostasis, which is essential for supporting neuronal health and meeting the diverse needs of highly plastic neuronal networks.^2^^,^^19^ We previously demonstrated that CHCHD10^S59L^ mutant induced dominant toxicity in *Drosophila* and HeLa cells, and that the PINK1/Parkin mediated pathway for maintaining mitochondrial quality and function was implicated in CHCHD10 associated ALS-FTD.^3^ Here, as a follow-up study, we demonstrate that FDA-approved PDE4 inhibitors successfully reduced morphological and functional mitochondrial defects in human cell lines and *in vivo Drosophila* model expressing CHCHD10^S59L^ and C2C10H^S81L^, respectively.

We observed that forskolin treatment reduced the Parkin and PINK1 accumulation in HeLa ^YFP−PARKIN^ and HeLa^PINK1-V5^ cells, respectively, after depolarization by an uncoupling agent, CCCP, as previously reported by Akabane and colleagues.^5^ Forskolin increased the cAMP levels of the system and activated the PKA signaling pathway, which led to the destabilization of PINK1 on depolarized mitochondria. Forskolin also reduced the disrupted mitochondrial network and enhanced mitochondrial respiration in transiently transfected CHCHD10^S59L^ HeLa cells. Similarly, the treatment of CHCHD10^S59L^ expressing cells with the PDE4 inhibitor roflumilast led to a partial rescue of the observed pathological phenotype. The mechanism by which these drugs act is the same, i.e., via the cAMP/PKA signaling pathway. This is achieved by increasing the cAMP levels in the cellular pool; one drug does this by hydrolyzing ATP to cAMP, and the other does this by inhibiting the hydrolysis of the phosphodiester bond of cAMP to AMP. The resulting activation of protein kinase A (PKA) modulates several downstream targets, including those involved in mitochondrial dynamics, particularly mitochondrial fusion, fission, and turnover.^4^ Mitochondria, being highly dynamic organelles, constantly undergo fusion and fission events to maintain their functional integrity. The disruption of this balance, often due to stress or disease condition, triggers quality control mechanisms, including mitophagy-the selective degradation of dysfunctional mitochondria. A key pathway governing mitophagy involves the accumulation of PINK1 and Parkin on the outer mitochondrial membrane of damaged mitochondria^20^ and also LC3, a ubiquitin-like protein that is covalently attached to the surface of an autophagosome during its biogenesis.^21^ CHCHD10^S59L^ overexpression caused LC3 accumulation in HeLa^YFP−Parkin^ cells as a result of active mitophagy via the PINK1/Parkin pathway. Both forskolin and roflumilast treatment led to a decrease in PINK1, Parkin, and LC3 accumulation in the cells. When specifically tested for mitophagy using the mitoQC system, Forskolin, Roflumilast, and Apremilast reduced the mitophagy flux, although Apremilast was not the most effective in reducing mitophagic flux compared to other drugs. Most effective recovery of energy metabolism likely also involves PINK1-dependent, Parkin-independent mechanisms, such as MIC60-dependent cristae organization, as we previously published.^3^ These findings highlight the complexity of mitochondrial quality control and underscore the need for further investigation to fully understand the relationship between bioenergetic enhancement and mitophagy regulation in the context of CHCHD10^S59L^-associated mitochondrial dysfunction. In our *Drosophila-*based model for ALS-FTD, we also report that forskolin and roflumilast significantly attenuate the C2C10H^S81L^-induced toxicity, highlighting their potential as modulators of mitochondrial stress *in vivo*.

PDE4 targeted therapies have shown promising outcomes in various neurological disorders such as Alzheimer’s disease (AD), Parkinson’s disease (PD), Fragile X Syndrome, depression, and neuropathic pain.^22^ However, the therapeutic armamentarium for ALS remains limited. One such drug, Ibudilast, a PDE4 inhibitor, is undergoing clinical trials for multiple neurodegenerative diseases, including Multiple Sclerosis and ALS due to its anti-inflammatory activity. In case of ALS, Ibudilast has been shown to slow the disease progression.^23^ Notably, Apremilast, another PDE4 inhibitor, is currently being evaluated in clinical trials and has been shown to cross the blood-brain barrier.^22^ In our experiments, apremilast has demonstrated strong effectiveness in reducing mitochondrial fragmentation and improving mitochondrial respiration at lower doses, outperforming both roflumilast and forskolin.

Our data suggest that PDE4 inhibitors may be effective therapeutics in the context of CHCHD10^S59L^-induced ALS-FTD. It also implies that the efficacy of PDE4 inhibitors for neurodegeneration may not only be a consequence of modulating neuroinflammation, as in the case of many previous clinical studies, but also regulating mitochondrial dynamics and homeostasis (which is the primary mechanism involved in CHCHD10^S59L^-associated ALS-FTD). Loss of TDP-43 function has been shown to result in the upregulation of phosphodiesterase 4 (PDE4) expression, suggesting a mechanistic link between TDP-43 dysfunction and altered cAMP signaling pathways.^24^ Consequently, it may be possible to increase the efficacy of PDE4 inhibitors against neurodegeneration by improving their ability to modulate mitochondrial biology rather than exclusively focusing on neuroinflammation. In fact, the combinatorial protective effect of forskolin and PDE4 inhibitors underscores the central role of cAMP turnover in regulating mitochondrial quality control. By simultaneously stimulating adenylate cyclase and inhibiting PDE4-mediated cAMP degradation, the combined treatment likely amplifies downstream signaling predominantly through PKA-dependent pathways, while achieving comparable protective effects at lower individual drug concentrations. This dual modulation strategy may therefore enhance efficacy while minimizing potential off-target or dose-related side effects. Considering that CHCHD10^S59L^ mutations disrupt mitochondrial dynamics and promote pathological mitophagy, combined cAMP modulation may represent a promising therapeutic strategy to restore mitochondrial function in CHCHD10-associated neurodegenerative disorders. In conclusion, our study endeavored to establish a framework for investigating PDE inhibitors as a potential candidate molecule in the context of CHCHD10-associated ALS-FTD. However, it is imperative to conduct rigorous preclinical and clinical studies to validate these effects and determine the most effective therapeutic regimen tailored to individual patients.

### Limitations of the study

While our findings support a role for PDE4 inhibition in reducing PINK1/Parkin accumulation and improving mitochondrial integrity, several limitations should be acknowledged. Although our data implicate the activation of the cAMP/PKA axis, the precise downstream effectors remain undefined. Potential plausible mechanisms, such as the PKA-mediated phosphorylation of mitochondrial proteins such as mitofilin (MIC60), or modulation of mitochondrial dynamics via AKAP1-anchored PKA inhibition of Drp1, require direct experimental confirmation.

Although our human cell lines and *Drosophila* model yielded consistent and concordant results, they have inherent constraints. Cell lines lack physiological complexity, whereas *Drosophila* does not fully reflect mammalian neuronal architecture. Therefore, validation in patient-derived iPSC models, organoids, and mammalian systems will be essential to strengthen translational relevance.

Finally, although PDE inhibitors have established clinical use, their safety, pharmacokinetics, and optimal dosing in the context of ALS-FTD are not well defined. Long-term effects, off-target consequences, and blood-brain barrier penetration require further evaluation. Additional preclinical studies will be necessary to determine whether targeting the cAMP/PKA pathway can be safely and effectively translated into therapeutic strategies.

## Resource availability

### Lead contact

Further information and requests for resources should be directed to the lead contact, Nam Chul Kim (kimn@umn.edu).

### Materials availability

This study did not generate any new unique reagents.

### Data and code availability

• All data reported in this article are available from the lead contact upon request.
• No original code is reported in this work.
• Additional information can be obtained from the lead contact upon request.

## Acknowledgments

This research was supported by grants from 10.13039/100000049NIA/10.13039/100000065NINDS R56NS112296 and Winston and Maxine Wallin Neuroscience Discovery fund R03NS149440 (to Nam Chul Kim). We thank Maddie Chalmers for her valuable feedback during the preparation of the article and Arpitaa Sharma for her assistance in obtaining data for the mitochondrial network analyses.

## Author contributions

S.M and D.H: data curation, methodology, investigation, formal analysis, writing – original draft, review, and editing. M.B: data curation, investigation, methodology, and formal analysis. Y-J.C: data curation, methodology, and investigation. N.C.K: conceptualization, supervision, funding acquisition, project administration, writing – original draft, review, and editing. All co-authors have read and approved the final version of the article.

## Declaration of interests

The authors declare no competing interests.

## STAR★Methods

### Key resources table

**Table:** 

REAGENT or RESOURCE	SOURCE	IDENTIFIER
**Antibodies**		

FLAG	Proteintech	Cat # 20543-1-AP; RRID:AB_11232216
FLAG	Sigma	Cat # F3165-1MG; RRID:AB_259529
TOM20 (D8T4N)	Cell Signaling Technology	Cat # 42406; RRID:AB_2687663
Pink 1	Novus Biologicals	Cat # BC100-494; RRID:AB_10127658
V5	Invitrogen	Cat # R96025; RRID:AB_2556564

**Chemicals, peptides, and recombinant proteins**		

CCCP	Sigma-Aldrich	Cat # C2759; CAS: 555-60-2
Forskolin	Millipore-Sigma	Cat # F3917-10MG; CAS: 66575-29-9
Roflumilast	Sigma-Aldrich	Cat # SML1099-5MG; CAS: 162401-32-3
Apremilast	MedChem Express	Cat # HY-12085/CS-0671; CAS:608141-41-9
Rolipram	Sigma-Aldrich	Cat # R6520-10MG; CAS: 61413-54-5
H-89 dihydrochloride hydrate	Sigma-Aldrich	Cat # B127; CAS: 130964-64-5

**Experimental models: Cell lines**		

HeLa	ATCC	CVCL_0030
HeLa-Parkin-YFP	Narendra D.P et al., 2010	–
Hela-Pink1-V5	Narendra D.P et al., 2010	–

**Experimental models: Organisms/strains**		

*Drosophila melanogaster*	Baek et al., 2021	MHC-GAL4, pUAST-CG5010 S81L- FLAG #2-3/TM2

**Recombinant DNA**		

Plasmid: pcDNA3-CHCHD10-S59L-FLAG	Baek et al., 2021	–
Plasmid: mCherry-LC3B	Addgene	Cat # 40827; Addgene_40827
Plasmid: mTagRFP-T-Mito-7	Addgene	Cat # 58023; Addgene_58023
Plasmid: Mito QC (pBabe.hygro-mcherry-GFP fis 101-152)	McWilliams et al., 2016	GenBank: NM_016068.2

**Software and algorithms**		

NIS-Element	Nikon	SCR_014329
Fiji ImageJ	Open	https://fiji.sc; SCR_002285
MiNA	Open	https://github.com/StuartLab; SCR_016137
SPSS	Open	SCR_019096

### Experimental model and study participant details

#### Animals

Transgenic *Drosophila* lines were generated by BestGene with the standard PhiC31 integrase-mediated transgenesis system.^3^ All transgenes were inserted into the third chromosome attp2 site to avoid positional gene expression differences. Fly cultures and crosses were performed on standard fly food (Genesee Scientific) and raised at 25°C with a 12:12 hr light: dark cycle. MHC-GAL4 was used as driver for the expression in the muscles. Both females and males flies were used in the experiments and data were analyzed sex-independently unless otherwise specified.

#### Cell culture

HeLa cells, HeLa^Parkin-YFP^, HeLa^PINK1-V5^ cells were gifts from Dr. Richard Youle’s lab.^11^ Cells were maintained in Dulbecco-modified Eagle medium (Gibco) supplemented with 10% fetal bovine serum (Gibco), 1× penicillin/streptomycin (Invitrogen), and 1 x GlutaMax Gibco). Cells were maintained in standard Cell Culture Facility. Cell line authentication was not independently performed by the authors. All cell lines were routinely checked for mycoplasma contamination assessed by DNA staining with DAPI and examined under a fluorescence microscope, and all cell lines tested negative.

### Method details

#### Plasmid constructs

The cDNA for human mutant form of CHCHD10 was synthesized and inserted in the pcDNA3 vector containing FLAG-tag by Genescripts. mTagRFP-T-Mito-7 (Plasmid #58023) and mCherryLC3B (#40827) plasmids were obtained from Addgene. pBabe.hygro-mcherry-GFP fis 101-152 (Mito QC) was a kind gift from Ian Ganley.

#### Cell transfections and drug treatments

Cells were transfected using jetPRIME (Polyplus #89129-924) transfection reagent according to the manufacturer’s protocol. Briefly, around 3X10^4^ cells were counted using ADAM-MC2 cell counter (NanoEntek) and plated in a 4-well chambered slides and allowed to grow for overnight. Transfections were carried out using 0.5 μg of plasmid and 1μl of jetPRIME transfection reagent (1:2) ratio and mixed in 50μL of jetPRIME buffer per 500ul of the cell culture media containing vehicle or respective drug (forskolin or PDE inhibitor). In cases of co-transfections, plasmids were mixed at equimolar concentrations (each of 0.25μg of plasmid). After 24 hrs of the transfection, cells were fixed and processed for immunostaining. For inhibitor studies, cells were pre-treated with the PKA inhibitor H89 (12.5 μM) for 4 hours before transfection and/or Forskolin or Roflumilast treatment to inhibit PKA activity.

#### Immunofluorescence and antibodies

Cells were fixed with 4% paraformaldehyde in 1X PBS for 10 mins, permeabilized with 0.1 % Triton X-100 for 10 mins and blocked with 5 % BSA in 1X PBS for 1 hr at room temperature. Primary antibodies were diluted in 5 % BSA in 1X PBS and slides were incubated overnight at 4°C or 2hrs at room temperature. Slides were then rinsed thrice with 1X PBST (0.1% tween-20) and incubated with secondary antibodies for 1.5 hrs at room temperature. After three washes with 1XPBST again, the slides were mounted with coverglass using Prolong Diamond Antifade Reagent with DAPI (Invitrogen; P36962) or Fluromount with DAPI (SouthernBiotech). Images were obtained with a LSM 710 (Zeiss, X63, 1.4 NA) confocal microscope or Nikon Crest X-light 2 spinning disc confocal microscope (Nikon). Co-localization was analyzed with ImageJ software. Primary antibodies used are FLAG (Sigma and Proteintech, 1:250), V5 (Life Technology, 1:200), PINK1 & TOM20 (Cell Signaling and Santa Cruz Biotechnology, 1:250) and Alexa Fluor-488, 594, 633 (Invitrogen) were used as secondary antibodies.

#### Mitochondrial respiratory activity assay

Mitochondrial respiration in HeLa, HeLa^Parkin-YFP^ cells were measured using the Seahorse Extracellular Flux Analyzer XFp (Agilent Technologies, #102416-100) with XF Cell Mito Stress Test Kit (Agilent Technologies, #103015-100). Approximately, 1 x 10^4^ transfected cells were plated into desired drug-containing media in V3-PS 96 well plate the day before performing an assay. Next day, media were replaced with fresh assay media supplemented with 1 mM pyruvate, 2 mM glutamine and 15 mM glucose with a pH adjusted to 7.6. The standard mitochondria stress tests were performed consisting of basal measurements followed by measurements after sequential addition of 1 μM oligomycin, 0.5 μM FCCP and 0.5 μM Rotenone/antimycin A. At the end of the assay, protein concentration of each well was determined using BCA assay to normalize the obtained OCR values.

#### Cyclic adenosine monophosphate (cAMP) assay

The levels of cAMP were detected in the cells by using a Direct cAMP Assay Kit (ab65355, Abcam) and was processed according to manufacturer’s protocol. Briefly, transfected cells were seeded into desired drug-containing media for 24 hrs. Next day added 100 μl/well of cell lysis buffer (0.1M of HCl) and incubated at room temperature for 20 minutes. Then, the lysates were centrifuged for 10 minutes at 13,000 rpm and the clear supernatant was extracted. Finally, the intracellular levels of cAMP were determined by measuring the absorbance at wave length of 450 nm using BioTek plate reader (Vermont, USA). The cAMP values were normalized to the protein concentration and expressed as pmol/mg protein.

#### Drug feeding

For drug feeding experiments, transgenic fly crosses were set in vials containing standard fly food supplemented with forskolin (50 μM), roflumilast (40 μM), and 0.1% DMSO.

#### Drosophila adult muscle preparation and immunohistochemistry

The sarcomere structure and mitochondrial morphology of the indirect flight muscle were prepared and analyzed as described here. Adult flies were anaesthetized with CO2 on a sleeping pad and fixed with 4% paraformaldehyde in 1X PBS for 1 hour after a quick dissection. They were then immersed in OCT compound (Fisher Scientific) and flash frozen in liquid nitrogen or dry ice. Fixed tissues (∼15-16 microns) were sectioned by a cryomicrotome (Leica) and directly mounted on the slide. Additional post fixing was performed with 4% paraformaldehyde in 1XPBS for 10 mins at room temperature and then permeabilized with 0.2% Triton X-100 in 1XPBS for 10 mins and blocked with 5% BSA solution for 1 hr. Sections were incubated with streptavidin-Alexa Fluor 488 and phalloidin–Alexa Fluor 594 (Invitrogen) overnight at 4°C for mitochondrial and muscle fiber staining respectively and mounted with Prolong Diamond Antifade Reagent with DAPI. Images were obtained with LSM 710 confocal microscope (Carl Zeiss) with 63X magnification and were processed with ImageJ. No consistent sex-dependent differences in sarcomere structure or mitochondrial morphology were observed under the experimental conditions examined.

#### Drosophila ATP assay

Fly thoraces (10-day-old male and female flies separately) were dissected out, collected and homogenized in 20 μl of homogenization buffer (100 mM Tris, 4 mM EDTA and 6 M guanidine-HCL, pH 7.8). The homogenate was centrifuged at 16,000 x g for 10 min and the supernatant was collected and diluted to 1:200 with deionized water for ATP measurement using the Cell Titer-Glo luminescent cell viability assay kit (Promega, G7571) and 1:10 for determining the protein concentration. Data was obtained by normalizing the ATP concentration with protein amount. No consistent sex-dependent differences in sarcomere structure or mitochondrial morphology were observed under the experimental conditions examined.

### Quantification and statistical analysis

For quantification of Parkin-YFP, PINK1, LC3 and Mitolysome accumulation in cells, a binary scoring method was employed: in each field of view, manual counting was done for the number of cells exhibiting accumulation (indicative of mitochondrial recruitment) versus those displaying diffuse cytoplasmic signal (indicative of no recruitment). These counts were then expressed as the percentage of cells with accumulation relative to the total number of cells in that field. Each experimental set include >200 cells for each group.

Mitochondrial length was measured using MiNA tool set combined with ImageJ software as previously described.^25^ In brief, this ImageJ plugin tool incorporates optional preprocessing steps to enhance the quality of images obtained from Confocal microscope. They were then converted to binary images and produced a morphological skeleton for calculating required parameters to quantitatively capture the morphology of the mitochondrial network.^11^ Data obtained from these were analyzed for statistical significance using one-way analysis of variance (ANOVA) followed by Dunnett’s *post-hoc* or Tukey’s *post-hoc* test. Data presentation was done in prism (Graphpad). Statistical significance relative to the DMSO control or untransfected control is indicated by asterisks (∗*p-value* < 0.05); (∗∗*p* < 0.01); (∗∗∗*p* < 0.001); (∗∗∗∗*p* < 0.0001). Cross symbols (†) denote statistical significance between indicated experimental groups.

Mitochondrial Respiration data were analyzed using one-way analysis of variance (ANOVA) followed by Dunnett’s *post-hoc* test to assess the differences relative to the control group. Statistical significance relative to the DMSO control or untransfected control is indicated by asterisks (∗*p-value* < 0.05); (∗∗*p* < 0.01); (∗∗∗*p* < 0.001); (∗∗∗∗*p* < 0.0001).

To quantify mitochondrial morphology in muscle tissue in *Drosophila*, all images were first standardized in ImageJ by applying the same color threshold settings. This ensured consistent detection of mitochondrial structures across all samples. After thresholding, the processed images were subjected to the ‘Analyze Particles’ function in ImageJ, which enabled the identification and quantification of individual mitochondrial particles based on size and shape parameters. For statistics, data obtained from these were analyzed using one-way analysis of variance (ANOVA) followed by Dunnett’s *post-hoc* test. Statistical significance relative to the DMSO control or untransfected control is indicated by asterisks (∗*p-value* < 0.05); (∗∗*p* < 0.01); (∗∗∗*p* < 0.001); (∗∗∗∗*p* < 0.0001).

## References

[1] Bannwarth S., Ait-El-Mkadem S., Chaussenot A., Genin E.C., Lacas-Gervais S., Fragaki K., Berg-Alonso L., Kageyama Y., Serre V., Moore D.G. (2014). A mitochondrial origin for frontotemporal dementia and amyotrophic lateral sclerosis through CHCHD10 involvement. Brain.

[2] Flippo K.H., Strack S. (2017). Mitochondrial dynamics in neuronal injury, development and plasticity. J. Cell Sci..

[3] Baek M., Choe Y.J., Bannwarth S., Kim J., Maitra S., Dorn G.W., Taylor J.P., Paquis-Flucklinger V., Kim N.C. (2021). TDP-43 and PINK1 mediate CHCHD10 S59L mutation–induced defects in Drosophila and *in vitro*. Nat. Commun..

[4] Merrill R.A., Dagda R.K., Dickey A.S., Cribbs J.T., Green S.H., Usachev Y.M., Strack S. (2011). Mechanism of neuroprotective mitochondrial remodeling by pka/akap1. PLoS Biol..

[5] Akabane S., Uno M., Tani N., Shimazaki S., Ebara N., Kato H., Kosako H., Oka T. (2016). PKA Regulates PINK1 Stability and Parkin Recruitment to Damaged Mitochondria through Phosphorylation of MIC60. Mol. Cell.

[6] Bondarev A.D., Attwood M.M., Jonsson J., Chubarev V.N., Tarasov V.V., Liu W., Schiöth H.B. (2022). Recent developments of phosphodiesterase inhibitors: Clinical trials, emerging indications and novel molecules. Front. Pharmacol..

[7] Newton A.C., Bootman M.D., Scott J.D. (2016). Second messengers. Cold Spring Harb. Perspect. Biol..

[8] Georgakopoulos N.D., Wells G., Campanella M. (2017). The pharmacological regulation of cellular mitophagy. Nat. Chem. Biol..

[9] Wedzicha J.A., Calverley P.M., Rabe K.F. (2016). Roflumilast: A review of its use in the treatment of COPD. International Journal of COPD.

[10] Kelly K., Mejia A., Suhasini A.N., Lin A.P., Kuhn J., Karnad A.B., Weitman S., Aguiar R.C.T. (2017). Safety and pharmacodynamics of the PDE4 inhibitor roflumilast in advanced B-cell malignancies. Clin. Cancer Res..

[11] Narendra D.P., Jin S.M., Tanaka A., Suen D.F., Gautier C.A., Shen J., Cookson M.R., Youle R.J. (2010). PINK1 is selectively stabilized on impaired mitochondria to activate Parkin. PLoS Biol..

[12] Jin S.M., Youle R.J. (2012). PINK1-and Parkin-mediated mitophagy at a glance. J. Cell Sci..

[13] Bauman A.L., Soughayer J., Nguyen B.T., Willoughby D., Carnegie G.K., Wong W., Hoshi N., Langeberg L.K., Cooper D.M.F., Dessauer C.W. (2006). Dynamic Regulation of cAMP Synthesis through Anchored PKA-Adenylyl Cyclase V/VI Complexes. Mol. Cell.

[14] Kim H.K., Hwang S.H., Oh E., Abdi S. (2017). Rolipram, a selective phosphodiesterase 4 inhibitor, ameliorates mechanical hyperalgesia in a rat model of chemotherapy-induced neuropathic pain through inhibition of inflammatory cytokines in the dorsal root ganglion. Front. Pharmacol..

[15] Sommer N., Löschmann P.A., Northoff G.H., Weller M., Steinbrecher A., Steinbach J.P., Lichtenfels R., Meyermann R., Riethmüller A., Fontana A. (1995). The Antidepressant Rolipram Suppresses Cytokine Production and Prevents Autoimmune Encephalomyelitis. Nat. Med..

[16] Allawh R., Hoffman M., Nadhan K.S., Abdelmalek M., Cusack C.A., Doyle A.M., Lee Chung C. (2020). Cutaneous disease in solid organ transplant recipients: A 5-year experience from a multidisciplinary medical-surgical transplant dermatology center. J. Am. Acad. Dermatol..

[17] McCann F.E., Palfreeman A.C., Andrews M., Perocheau D.P., Inglis J.J., Schafer P., Feldmann M., Williams R.O., Brennan F.M. (2010). Apremilast, a novel PDE4 inhibitor, inhibits spontaneous production of tumour necrosis factor-alpha from human rheumatoid synovial cells and ameliorates experimental arthritis. Arthritis Res. Ther..

[18] Zhao J., Wang X., Huo Z., Chen Y., Liu J., Zhao Z., Meng F., Su Q., Bao W., Zhang L. (2022). The Impact of Mitochondrial Dysfunction in Amyotrophic Lateral Sclerosis. Cells.

[19] Aslam M., Ladilov Y. (2021). Regulation of mitochondrial homeostasis by sac-derived camp pool: Basic and translational aspects. Cells.

[20] Pickles S., Vigié P., Youle R.J. (2018). Mitophagy and Quality Control Mechanisms in Mitochondrial Maintenance. Curr. Biol..

[21] Gong G., Zhang H., Kolwicz S.C. (2023). Editorial: Mitochondrial control of cell fate. Front. Cell Dev. Biol..

[22] Crocetti L., Floresta G., Cilibrizzi A., Giovannoni M.P. (2022). An Overview of PDE4 Inhibitors in Clinical Trials: 2010 to Early 2022. Molecules.

[23] Oskarsson B., Maragakis N., Bedlack R.S., Goyal N., Meyer J.A., Genge A., Bodkin C., Maiser S., Staff N., Zinman L. (2021). MN-166 (ibudilast) in amyotrophic lateral sclerosis in a Phase IIb/III study: COMBAT-ALS study design. Neurodegener. Dis. Manag..

[24] Briese M., Saal-Bauernschubert L., Lüningschrör P., Moradi M., Dombert B., Surrey V., Appenzeller S., Deng C., Jablonka S., Sendtner M. (2020). Loss of Tdp-43 disrupts the axonal transcriptome of motoneurons accompanied by impaired axonal translation and mitochondria function. Acta Neuropathol. Commun..

[25] Valente A.J., Maddalena L.A., Robb E.L., Moradi F., Stuart J.A. (2017). A simple ImageJ macro tool for analyzing mitochondrial network morphology in mammalian cell culture. Acta Histochem..

