# Supplementary material for: FDA-approved PDE4 inhibitors alleviate the dominant toxicity of ALS-FTD-associated CHCHD10S59L in Drosophila and human cells
Source: iScience. 2026 Feb 2;29(3):114879. doi: 10.1016/j.isci.2026.114879 (PMC12925567; doi:10.1016/j.isci.2026.114879)
Supplement: Document S1. Figures S1–S5 [file mmc1.pdf]

iScience, Volume *29*

Supplemental information

FDA-approved PDE4 inhibitors alleviate the dominant toxicity of ALS-FTD-associated CHCHD10^{S59L} in *Drosophila* and human cells

Swati Maitra, Do-Won Ham, Minwoo Baek, Yun-Jeong Choe, and Nam Chul Kim

Figure S1

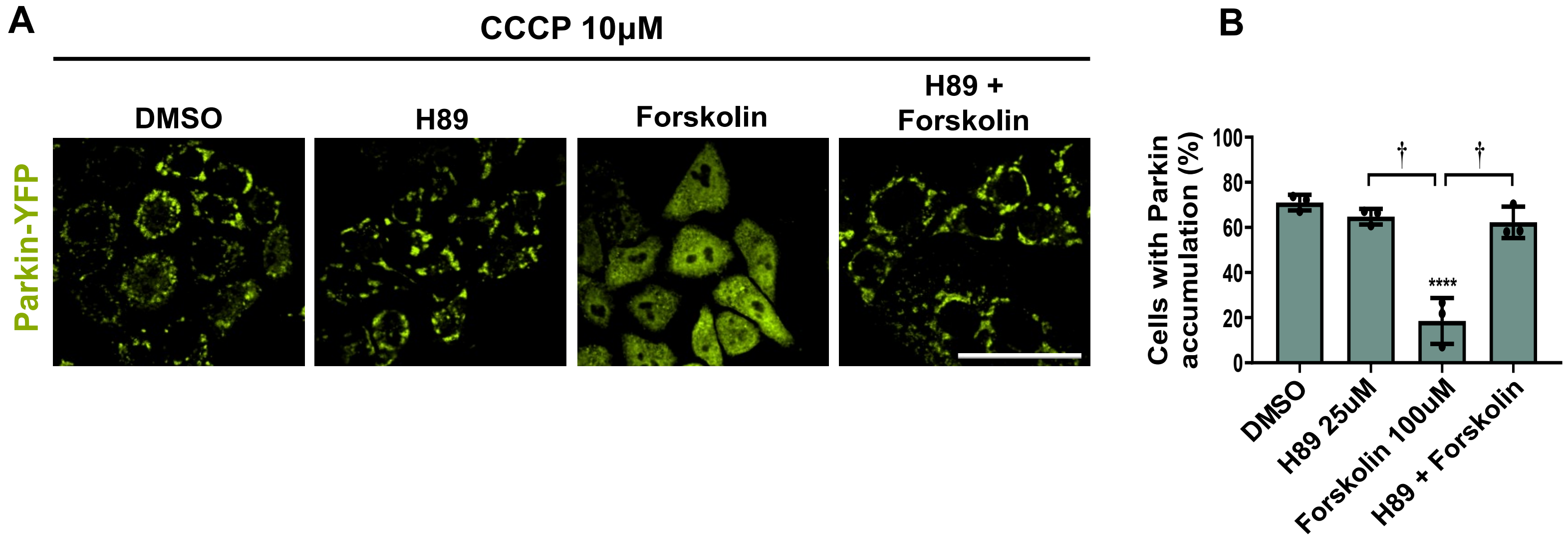

A
CCCP 10μM
DMSO
H89
Forskolin
H89 + Forskolin
Parkin-YFP
B
Cells with Parkin accumulation (%)
100
80
60
40
20
0
†
†
****
DMSO
H89 25uM
Forskolin 100uM
H89 + Forskolin

Supplementary Figure S1. Forskolin suppresses CCCP-induced Parkin accumulation through cAMP–PKA signaling in HeLa$^{\text{Parkin-YFP}}$ cells.

(A) Representative confocal images of HeLa$^{\text{Parkin-}}$$_{\text{YFP}}$ cells treated with CCCP (10 μM-1hr) pretreated with DMSO, H89 (25 μM-4hr), forskolin (100 μM-2hr), or a combination of H89 and forskolin. **(B)** Quantification of cells showing Parkin accumulation based on the images in (A). Forskolin treatment markedly reduced Parkin puncta formation, while PKA inhibition by H89 abrogated this effect. Scale bar, 50 μm. Data are presented as mean ± SD (one-way ANOVA and post hoc Tukey's Test, ****$p < 0.0001$ vs. DMSO, †$p < 0.05$, between indicated groups).

Figure S2

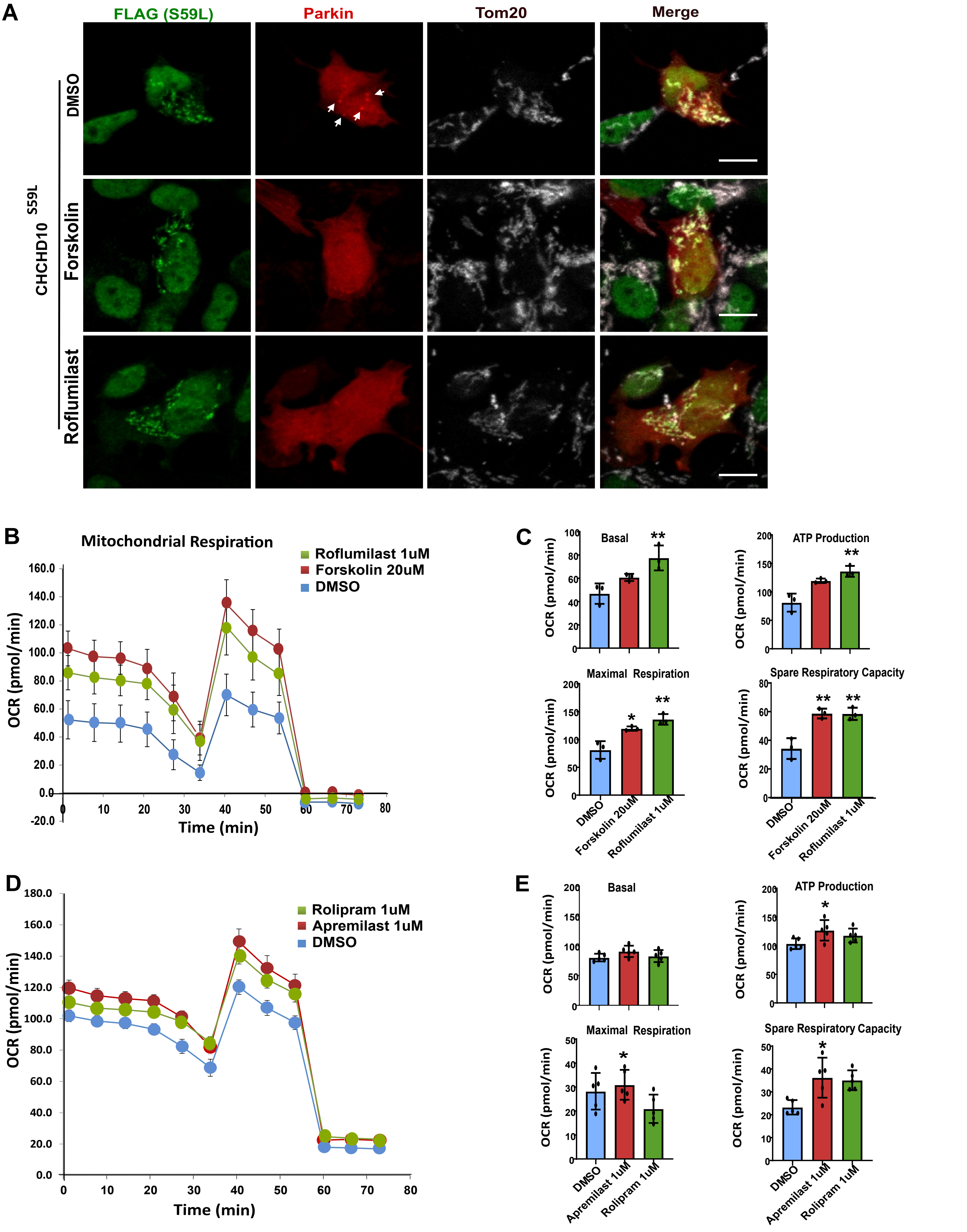
A
FLAG (S59L)
Parkin
Tom20
Merge
DMSO
Forskolin
Roflumilast
CHCHD10 S59L
B
Mitochondrial Respiration
Roflumilast 1uM
Forskolin 20uM
DMSO
OCR (pmol/min)
Time (min)
C
Basal
ATP Production
Maximal Respiration
Spare Respiratory Capacity
DMSO
Forskolin 20uM
Roflumilast 1uM
D
Rolipram 1uM
Apremilast 1uM
DMSO
OCR (pmol/min)
Time (min)
E
Basal
ATP Production
Maximal Respiration
Spare Respiratory Capacity
DMSO
Apremilast 1uM
Rolipram 1uM

Supplementary Figure S2. PDE inhibition attenuates CHCHD10^{S59L}-associated Parkin accumulation and mitochondrial respiratory defects in SH-SY5Y cells.

SH-SY5Y cellswere co-transfected with FLAG-tagged CHCHD10^{S59L} and Parkin-mCherry in DMSO, Forskolin, and Roflumilast containing medium. **(A)** Representative images from CHCHD10^{S59L} expressing mitochondria of SHSY5Y cells cultured in DMSO, forskolin (50 μM-1hr) and roflumilast (10 μM-3hr) containing media. Cells were immunostained against anti-FLAG (green, CHCHD10^{S59L}) and anti-TOM20 (white, mitochondria) antibodies. Arrows indicated accumulated parkin in the CHCHD10^{S59L} expressing mitochondria. **(B-C)** Mitochondrial respiration was measured by Seahorse XF Cell Mito Stress tests 24hrs after CHCHD10^{S59L} transfection in DMSO, forskolin (20 μM) and roflumilast (1 μM) treated SHSY5Y cells. **(D-E)** Mitochondrial respiration by Seahorse XF Cell Mito Stress tests 24hrs after CHCHD10^{S59L} transfection in DMSO, apremilast (1 μM) and rolipram (1 μM) treated SHSY5Y cells. Data are shown as mean ±SD (one-way ANOVA and *post hoc* Dunnett test, two-sided, comparison with DMSO, ***$p < 0.01$ and *$p < 0.05$).

Figure S3

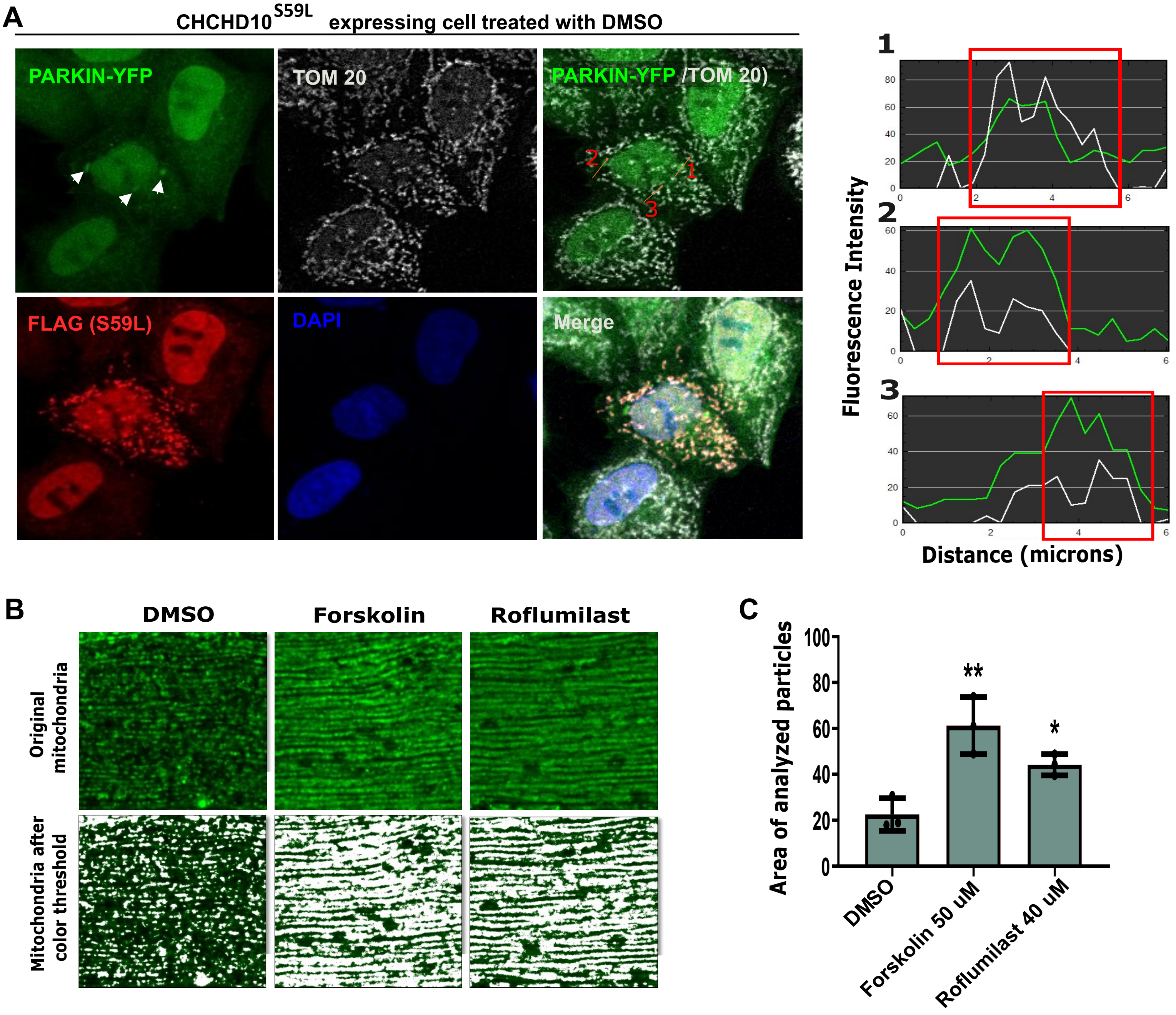
A
CHCHD10 S59L expressing cell treated with DMSO
PARKIN-YFP
TOM 20
PARKIN-YFP /TOM 20)
FLAG (S59L)
DAPI
Merge
1
2
3
Fluorescence Intensity
Distance (microns)
B
DMSO
Forskolin
Roflumilast
Original mitochondria
Mitochondria after color threshold
C
Area of analyzed particles
**
*
DMSO
Forskolin 50 uM
Roflumilast 40 uM

Supplementary Figure S3. Forskolin and roflumilast reduce CHCHD10^{S59L}-associated Parkin accumulation and mitochondrial abnormilities in cells and *Drosophila* muscle.

(A) Colocalization analysis of representative images of CHCHD10^{S59L} expressing HeLa$^{PARKIN-YFP}$ cells cultured in DMSO (from Figure 5A). Cells were immunostained against anti-FLAG (red, CHCHD10^{S59L}) and anti-TOM20 (white, mitochondria) antibodies. Arrows indicated sites of accumulated parkin in the CHCHD10^{S59L} expressing mitochondria. **(B)** Representative images used for mitochondrial quantification in the indirect flight muscles of 10-day-old adult flies (MHC-Gal4 > UAS-S81L) treated with DMSO, Forskolin (50μM), or Roflumilast (40μM). First panel: immunostaining with streptavidin-Alexa Fluor 488 (mitochondria) and phalloidin-Alexa Fluor 594 (actin). Second panel: images processed with a standardized color threshold applied uniformly across all treatment groups. **(C)** Quantitative analysis showing the area of mitochondrial particles in indirect flight muscles of flies treated with DMSO, Forskolin, or Roflumilast, based on ImageJ analysis (n=3). Data are shown as mean ±SD (one-way ANOVA followed by *post hoc* Dunnett test, comparison with DMSO, $^{**}p < 0.01$ and $^{*}p < 0.05$).

Figure S4

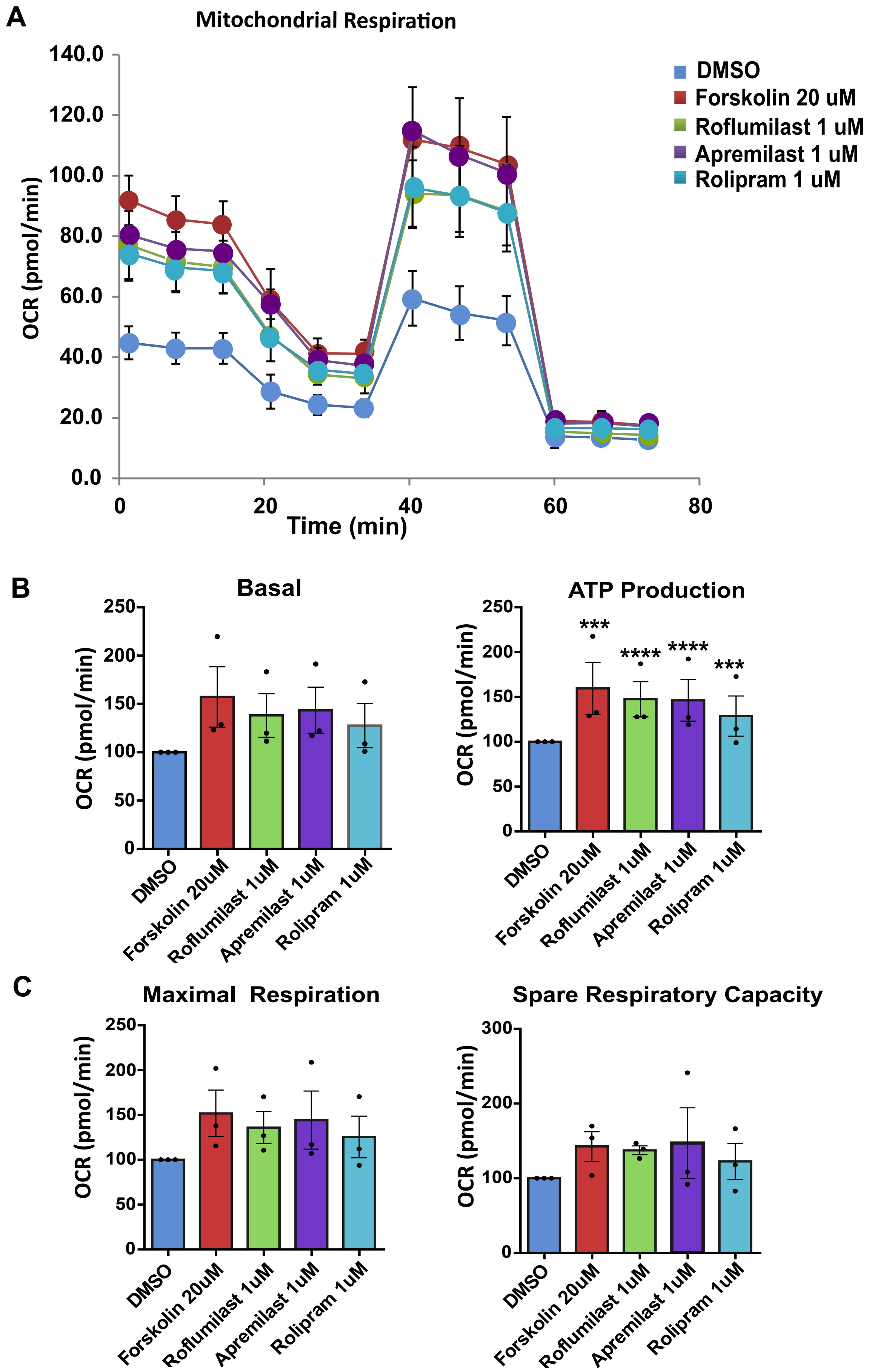
A
Mitochondrial Respiration
OCR (pmol/min)
Time (min)
DMSO
Forskolin 20 uM
Roflumilast 1 uM
Apremilast 1 uM
Rolipram 1 uM
B
Basal
ATP Production
***
****
****
***
OCR (pmol/min)
DMSO
Forskolin 20uM
Roflumilast 1uM
Apremilast 1uM
Rolipram 1uM
C
Maximal Respiration
Spare Respiratory Capacity

Supplementary Figure S4. PDE inhibitors restore mitochondrial respiratory function impaired by CHCHD10^{S59L} expression in HeLa cells.

(A) Mitochondrial respiration was measured by Seahorse XF Cell Mito Stress tests 24hrs after CHCHD10^{S59L} transfection in DMSO, forskolin, roflumilast, apremilast and rolipram treated HeLa cells. **(B) (C)** Bar graphs indicating basal respiration, ATP production, Maximal respiration, Spare respiratory capacity measured during seahorse assay in HeLa cells after CHCHD10^{S59L} transfection in DMSO, forskolin, roflumilast, apremilast and rolipram treatment. The data presented here shows the overall comparison of all 4 drugs from 3 independent experiments. Data shown here is normalized to DMSO and expressed as a percentage with mean ±SD (one-way ANOVA and *post hoc* Dunnett test, two-sided, comparison with DMSO, ****$p < 0.001$, ***$p < 0.01$, and *$p < 0.05$).

Figure S5

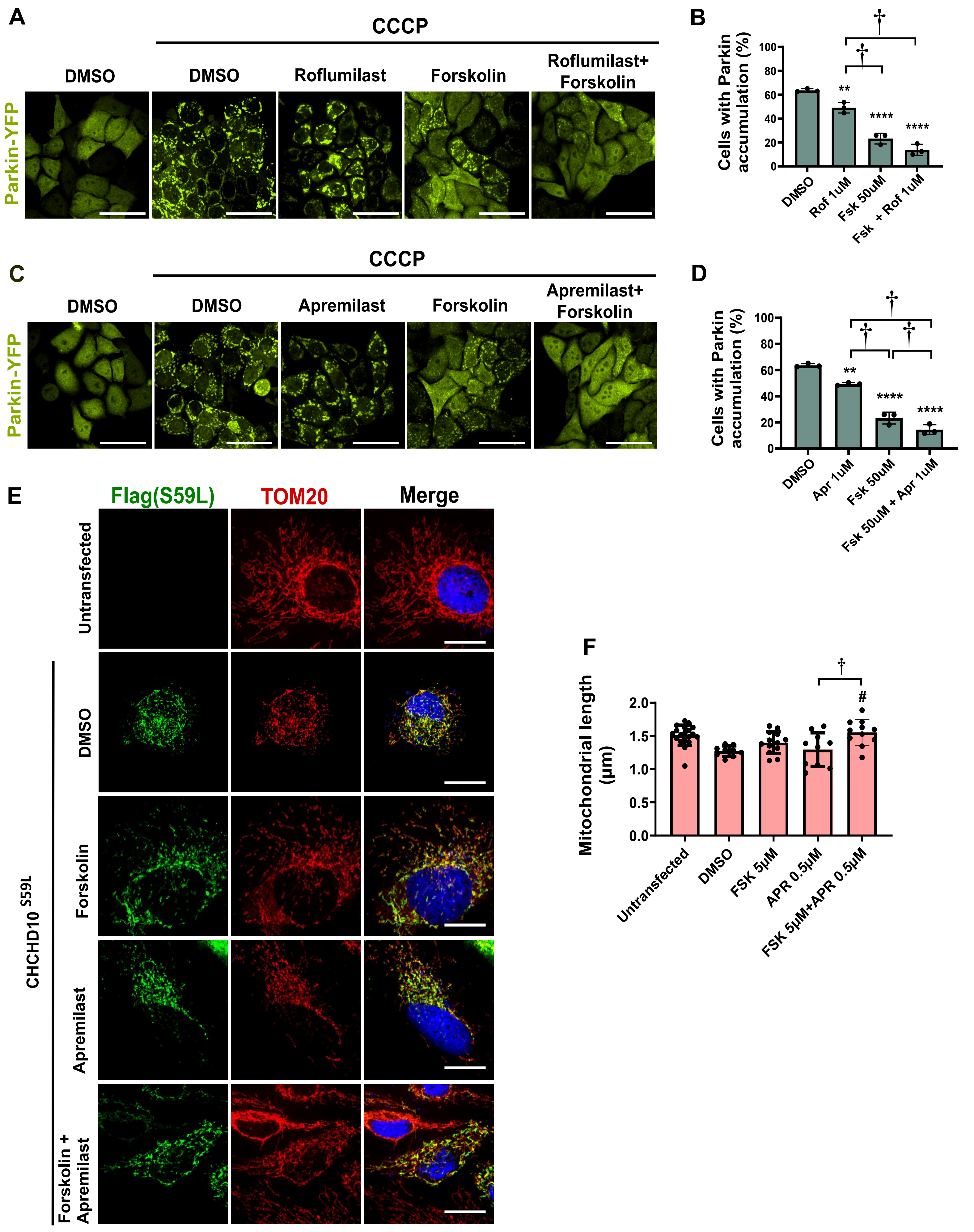

A
CCCP
DMSO
DMSO
Roflumilast
Forskolin
Roflumilast+
Forskolin
Parkin-YFP
B
Cells with Parkin accumulation (%)
100
80
60
40
20
0
**
****
****
DMSO
Rof 1uM
Fsk 50uM
Fsk + Rof 1uM
C
CCCP
DMSO
DMSO
Apremilast
Forskolin
Apremilast+
Forskolin
Parkin-YFP
D
Cells with Parkin accumulation (%)
100
80
60
40
20
0
**
****
****
DMSO
Apr 1uM
Fsk 50uM
Fsk 50uM + Apr 1uM
E
Flag(S59L)
TOM20
Merge
Untransfected
DMSO
Forskolin
Apremilast
Forskolin + Apremilast
CHCHD10 S59L
F
Mitochondrial length (µm)
2.0
1.5
1.0
0.5
0.0
#
Untransfected
DMSO
FSK 5µM
APR 0.5µM
FSK 5µM+APR 0.5µM

Supplementary Figure S5. Combined effect of forskolin and PDE4 inhibitors on Parkin accumulation and mitochondrial integrity in CHCHD10^S59L-expressing HeLa cells. $HeLa^{Parkin\text{-}YFP}$ cells were pre-treated with Forskolin, PDE4 inhibitors or both followed by CCCP. PDE4 inhibitors were treated (1 μM-24hrs), Forskolin was treated (50 μM-2hrs) and CCCP was treated (10 μM-1hr). **(A)** Representative confocal images of $HeLa^{Parkin\text{-}YFP}$ cells showing mitochondrial Parkin accumulation in DMSO, CCCP, Forskolin + CCCP, Roflumilast + CCCP, and Forskolin + Roflumilast + CCCP treatment groups. Scale bar = 50 μm. **(B)** Quantification of cells showing mitochondrial Parkin-YFP accumulation across each group corresponding to the images shown in (A) (n = 3 independent replicates; 200–300 cells per group). **(C)** Representative images showing mitochondrial Parkin-YFP accumulation in DMSO, CCCP, Forskolin + CCCP, Apremilast + CCCP, and Forskolin + Apremilast + CCCP groups. Scale bar = 50 μm. **(D)** Quantification of cells showing mitochondrial Parkin-YFP accumulation across each group corresponding to the images shown in (C) (n = 3 independent replicates; 200–300 cells per group). Data are presented as mean ± SD (one-way ANOVA followed by *post hoc* Tukey's test; ***$p < 0.001$, **$p < 0.01$, *$p < 0.05$). An asterisk indicates a significant difference compared with the DMSO (non-CCCP) group, while a cross (†) indicates significant differences between treatment groups ($p < 0.05$). HeLa cells were transfected with FLAG-tagged $CHCHD10^{S59L}$ and treated with Forskolin (5 μM) and Apremilast (0.5 μM) or both for 24 h. **(E)** Representative images of cells immunostained with anti-FLAG (green, $CHCHD10^{S59L}$), anti-TOM20 (Red, mitochondria), and DAPI (blue, nuclei). Scale bar = 20 μm. **(F)** Quantification of mitochondrial length. Combined treatment with Forskolin and Apremilast significantly restored mitochondrial network integrity compared with Apremilast (0.5 μM) treatments

alone ($p < 0.05$, one-way ANOVA followed by *post hoc* Tukey's test). Data are presented as mean ± SD (# $p < 0.05$ vs. DMSO; † $p < 0.05$ between indicated groups.).
